# Development of a Sensory Lexicon and Predictive ANN Modeling for Black Queen Wine: A Novel Workflow Incorporating Bridge-Linked QDA and Consumer Hedonic Analysis

**DOI:** 10.3390/foods15122158

**Published:** 2026-06-15

**Authors:** Gus Chang-Hung Han, Shuo-Wen Tsai

**Affiliations:** Department of Food Science and Biotechnology, National Chung Hsing University, Taichung 40227, Taiwan; d107043003@mail.nchu.edu.tw

**Keywords:** Black Queen wine, sensory lexicon, Artificial Neural Network (ANN), data bridging, multicollinearity, non-linear interaction, subtropical viticulture

## Abstract

*Vitis vinifera* L. *× Vitis labrusca* L. cv. Black Queen (BQ) is a hybrid cultivar with oenological potential in subtropical climates, yet its sensory structure remains insufficiently systematized. This study aimed to construct an integrated sensory framework by merging two Balanced Complete Block Design (BCBD) datasets into a unified database and developing a structured descriptor reduction workflow to address multicollinearity and redundancy. The resulting “BQ Lexicon v.0” comprised nine Quantitative Descriptive Analysis (QDA) attributes and twelve check-all-that-apply (CATA) descriptors. Based on this optimized dataset, an Artificial Neural Network (ANN) model was developed to predict overall liking (OL), achieving a satisfactory performance (*R*^2^(train) = 0.70 and *R*^2^(validation) = 0.74). Three-dimensional response surface visualization further illustrated non-linear relationships as a process monitor, indicating sourness as a primary negative driver of acceptance and revealing interactive and synergistic effects between tannin, sweetness, and aroma. These findings demonstrate that integrating structured data management with machine learning can enhance sensory modeling efficiency. Ultimately, the validated BQ Lexicon v.0 and the aligned data framework establish a reliable foundation for future oenological research in Black Queen grape. This structured approach effectively resolves the challenges of integrating distributed sensory datasets, while offering practical insights for targeted winemaking strategies.

## 1. Introduction

Wine holds a unique position in food science, characterized by a sophisticated production tradition that contrasts sharply with modern food-processing techniques such as extraction, fortification, or flavoring [1]. The oenological process remains predominantly reliant on complex biological phenomena, including maceration, microbial metabolism, and yeast autolysis [2], all governed by stringent regulatory frameworks [3]. Historically, the fundamental methodologies of winemaking and tasting have remained remarkably consistent over centuries, allowing for the accumulation of an extensive longitudinal scientific record [4]. Since the seminal work of Louis Pasteur (1822–1895), wine has been the subject of comprehensive documentation spanning both “Old-World” and “New-World” regions. Although categorized as a cash crop or horticultural crop, the research depth and scale associated with viticulture and oenology are unparalleled among non-staple food products [4].

In the domain of sensory science and mathematical modeling, traditional wine evaluation has historically leaned on classical multivariate statistical approaches and linear models such as PCA and PLS [5]. This reliance necessitates experimental designs such as Balanced Complete Block Designs (BCBDs), which, despite their rigor, are labor-intensive and yield a limited sample space that fails to meet the data-intensive requirements of modern deep learning and artificial intelligence algorithms [6,7]. Furthermore, these traditional frameworks operate under the assumption that sensory attributes are linearly independent, with minimal or negligible interactions [8,9]. While such models suffice for basic trend analysis within limited scales (e.g., five-point Likert or 100 mm unstructured line scales) using validated descriptors, they often overlook critical non-linear nuances and synergistic effects essential for high-precision modeling [10].

The present study introduces a methodological innovation by utilizing a two-block sensory design [11]. This approach not only mitigates the pervasive issue of sensory fatigue—a common limitation in traditional Quantitative Descriptive Analysis (QDA)—but also establishes a robust empirical foundation for the integration of Artificial Neural Networks (ANNs) [10]. Such methodological rigor aligns with the contemporary shift towards integrating sensory science with advanced data science applications.

To broaden the global sensory and wine landscape, this research focuses on a neglected but significant hybrid cultivar: the Black Queen (BQ) grape (*Vitis vinifera L. × Vitis labrusca L.* cv. Black Queen) [12,13,14]. Developed for its resilience to high humidity and temperatures, the Black Queen grape is a cornerstone of subtropical viticulture in East Asia, yet its sensory profile remains inadequately characterized. This study also utilizes international benchmark varieties (Cabernet Sauvignon and Syrah) as “bridges” to normalize data across independent blocks. By assessing consensus through *p*-values and coefficients of variation (CVs), we initiate the construction of a large-scale sensory database specifically for this cultivar.

The Black Queen wine is distinguished by its high acidity, deep pigmentation and intense aromatic profile [12]. To establish a globally compatible framework, this research develops the “BQ Lexicon v.0.” This process involves screening attributes based on frequency, followed by Principal Component Analysis (PCA) and Correspondence Analysis (CA) to eliminate semantic redundancies and tautologies [15]. The resulting lexicon, comprising nine QDA and 12 CATA (check-all-that-apply) traits, was further validated through consumer-based penalty analysis (mean drop).

Finally, this study applies ANN modeling to decode the complex, non-linear relationships and hidden interaction terms within the sensory data. Through the visualization of 3D response surfaces, we demonstrate the robustness of the model and its potential as a reliable reference for industrial process optimization. The uniqueness of this research lies in its exploration of a rare subtropical variety, the implementation of bridge-data strategies for deep learning, and successful improvement of model transparency through feature importance analysis.

## 2. Materials and Methods

This study employed six wine samples, evaluated by 22 trained panelists. Initially, 52 CATA descriptors and QDA sensory intensity scores were collected from the WSET, CMS and Arom kit vocabulary list. After statistical screening, redundancy reduction and panel review, 21 sensory attributes were retained in a lexicon specified for Black Queen, and 18 sensory attributes were used as ANN input variables to monitor the process and predict overall liking (OL).

### 2.1. Plant Material and Wine Samples

The specified wine samples evaluated in this study were produced from the Black Queen grape (*Vitis vinifera* L. *× Vitis labrusca* L. cv. Black Queen), a hybrid cultivar developed by Zenbai Kawakami (1868–1944) [14]. Four variants of Black Queen (BQ) wines (labeled as BQ-A, BQ-B, BQ-C, and BQ-D) were analyzed alongside two international benchmark varieties—Cabernet Sauvignon (CAB) and Syrah (SYR)—which served as sensory anchor points for data bridging [11], totaling six wine samples. All Black Queen crops were hand-harvested in the 2022 and 2023 summer and winter in Erlin Township, Changhua County, Taiwan. All wine samples were stored at a controlled temperature (18 ± 2 °C) and followed the ISO 3591 standard [16], using 215 mL wine-tasting glasses, each coded with a random three-digit number to eliminate bias.

### 2.2. Sensory Panels, Experimental Design, and Ethical Considerations

The sensory evaluation procedures were conducted in strict accordance with the Declaration of Helsinki. This study involved non-invasive sensory testing of food-grade, commercially available wine samples by healthy adult volunteers. All participants provided written informed consent, which included a commitment to moderate alcohol consumption and confirmation of legal drinking age. To ensure privacy, all data were fully anonymized using unique identifier codes, and no personal identifying information was recorded. Given the low-risk nature of this study and the use of standard food safety protocols, this research met the criteria for exemption from formal institutional review board approval.

Sensory profiling was executed using two distinct panels. First, Quantitative Descriptive Analysis (QDA) was conducted using a trained panel of 22 assessors across two independent experimental blocks to mitigate sensory fatigue and facilitate large-scale database integration. Both blocks utilized a Balanced Complete Block Design (BCBD) to ensure statistical validity. Second, a consumer panel consisting of 74 individuals participated in the hedonic test to evaluate acceptance scores, generating a total of 444 assessments (74 consumers × 6 wines).

### 2.3. Data Preprocessing and Lexicon Development

**Figure 1:**
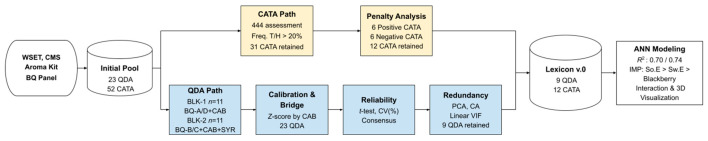
Methodological framework for the establishment and validation of the Black Queen sensory lexicon.

As illustrated in the overarching methodological framework (Figure 1), The yellow boxes represent the CATA path, while the blue boxes indicate the QDA path. This research was executed through three interconnected sequential modules: structured data acquisition and dataset bridging, dimensionality reduction and Lexicon v.0 development, and predictive modeling via Artificial Neural Networks (ANNs). The initial phase focused on capturing both objective QDA intensity profiles and subjective CATA consumer responses, utilizing a benchmark bridge sample strategy to systematically align the distributed evaluation blocks. Following the alignment, the workflow transitioned into a systematic data reduction phase to filter out tautological redundancy and multicollinearity. Once the streamlined BQ Lexicon v.0 was established, the framework’s final module utilized these core sensory attributes as input variables for non-linear predictive modeling. This integrated architecture was specifically designed to translate high-dimensional sensory matrices into robust, actionable process benchmarks.

Data cleaning and preliminary statistical preprocessing were performed using Python 3.13. The establishment of the “BQ Lexicon v.0” followed a multi-stage data management workflow. To ensure model interpretability and avoid multicollinearity, Principal Component Analysis (PCA) and Correlation Analysis were employed to identify redundant factors and address the issue of data redundancy. Sensory attributes with high cross-loadings or consistent grouping within the same principal component (rotated factor loading > 0.50) were consolidated. XLSTAT 2019 was utilized for Penalty Analysis (mean drop) to identify the “must-have” and “must-not-have” attributes influencing OL.

### 2.4. Statistical Analysis and Artificial Neural Network (ANN) Modeling

The variance inflation factor (VIF) was calculated for all predictors, and only traits with VIF values below 3.0 were retained to ensure independent feature information. Correspondence Analysis (CA) mapped the sensory positions of BQ variants relative to the bridge samples (CAB and SYR), with differences assessed via Cochran’s Q test (*p* < 0.05). To predict consumer acceptance based on the refined lexicon, an Artificial Neural Network (ANN) predictive model was implemented using JMP Pro 18 (SAS Institute, Cary, NC, USA). A multilayer perceptron (MLP) structure with a single hidden layer was developed to model the non-linear relationship between sensory attributes and consumer OL. Following the Parsimony Principle to reduce the overfitting risk from the beginning, 18 independent variables were selected as predictors. These comprised 7 objective intensity attributes from QDA (RtP.E, Aro.E, Sw.E, So.E, Oak.E, Tan.E, and Bit.E) and 11 selected CATA terms (blackberry, red berry, alcohol, violet, red plum, lactic acid, hay, tropical fruit, moldy, sulfur dioxide, and meaty). All inputs were standardized using *Z*-score transformation to prevent gradient saturation, and the informative missing option was disabled (0). Hidden Layer and Activation: The hidden layer structure was configured with 2 hyperbolic tangent (TanH) and 4 Gaussian activation functions. The inclusion of Gaussian functions allowed the model to effectively capture non-monotonic “ideal-point” behaviors without requiring manual quadratic expansions. The network was trained using a Robust Fit approach (Robust Fit = 1) and a squared penalty method (weight decay) to minimize outlier bias. The training process utilized 80 random tours and 40 boosts to optimize architecture convergence. The model was validated using 5-fold cross-validation (K-fold) with a fixed random seed (888). The final model was selected based on maximizing *R*^2^ and minimizing RMSE. To maintain ecological validity, non-axis input variables were frozen at their empirical sample means derived from the global consumer database (*n* = 444), restricting evaluations to the structural centroid of the Black Queen wine sensory space and preventing extrapolation artifacts.

## 3. Results

Sensory data bridging via Cabernet Sauvignon proved highly effective, with independent *t*-tests showing no significant differences between two evaluation blocks. Intra-block consistency categorized attributes into three consensus levels, with Block 2 showing lower coefficient of variation (CV) values likely due to panel familiarity with two international cultivars (CAB and SYR). To consolidate the final lexicon, Principal Component Analysis and Cluster Analysis successfully streamlined synonymous descriptors, yielding nine Quantitative Descriptive Analysis (QDA) and 12 check-all-that-apply (CATA) attributes after expert panel review and Penalty Analysis. The subsequent Artificial Neural Network (ANN) achieved satisfactory predictive power (*R*^2^ > 0.70), identifying sourness as a dominant factor requiring enological reduction. High Total-to-Main Effect ratios for sourness (5.04), sweetness, and aroma confirmed that non-linear interaction effects far outweighed linear direct effects, as visually validated by the highly complex topography of the 3D response surface plots and a narrow process window.

### 3.1. Data Bridging via Reference Cabernet Sauvignon

To ensure the compatibility of sensory data collected across different sessions and to facilitate future data fusion for deep learning applications, an inter-block consistency analysis was performed using a reference “bridge” sample (HARDY’S Cabernet Sauvignon, 5 L BIB). The data from Block 1 (*n* = 11) and Block 2 (*n* = 11) were first subjected to Global Normalization using Z-scores to mitigate individual scale usage bias. Independent *t*-tests conducted on the normalized mean scores of 23 sensory attributes revealed no significant differences (*p* > 0.05) between the two blocks for all descriptors (Table 1). No statistical significance indicates that the mean intensities perceived by both block panels were aligned. To further evaluate the consensus within and between the blocks, the coefficient of variation (CV) was employed with a threshold of 30%, 10% wider than the normal width, in accordance with the BQ rarity and unfamiliarity [17,18].

The 23 attributes were categorized into three groups based on their consensus status:

- High consensus (10 attributes): Descriptors including appearance (Dep.E; RtP.E), aroma (Aro.E; Fru.E), taste (So.E), and mouthfeel/general (Tan.E, Cor.E, Alc.E, Bal.E, and Com.E) exhibited CV values below 30%. These attributes showed high reliability and are considered the core sensory framework for the Black Queen lexicon.
- Low consensus (six attributes): Attributes such as Flo.E, Spi.E, Her.E, Veg.E, Aft.E, and Sw.E showed CV values exceeding 30%, indicating higher intra-group variance despite the non-significant *t*-test results.
- Baseline noise (seven attributes): Descriptors including Fla.E, Dry.E, Car.E, Ani.E, Min.E, Oak.E, and Bit.E were classified as noise. These attributes were characterized by low mean intensities (mean < 3.0), which mathematically inflated the CV values (>80%), representing sub-threshold sensory signals rather than a lack of panelist competence.

### 3.2. Principal Component Analysis and Feature Selection

**Figure 2:**
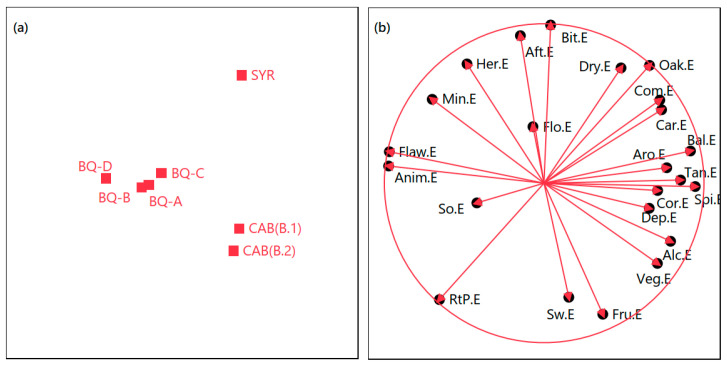
PCA of sensory attributes for Black Queen and reference wine samples: (**a**) 6 wine samples; (**b**) 23 QDA sensory attributes.

To characterize the product space and evaluate the relationships between sensory attributes, Principal Component Analysis (PCA) was performed on the mean scores of the 23 descriptors (Figure 2). The first two principal components accounted for 73.4% of the total variance, providing a good and acceptable representation of the sensory dynamics (Figure 2). PC1 (44.7%—Structure and Intensity Axis): This dimension was anchored on the positive end by attributes representing wine structure and aromatic intensity, such as balance (Bal.E), tannin (Tan.E), spicy (Spi.E), and overall aroma (Aro.E). The negative end was dominated by flaw (Fla.E) and animal (Ani.E). Product Mapping: A clear segregation was observed between the international “bridge” samples and the Black Queen (BQ) wines. CAB (B.1, B.2), from each design block, and SYR were positioned on the far right of PC1, reflecting superior structural complexity. In contrast, all BQ samples (A–D) clustered on the negative side, highlighting a distinct “varietal/fermentation identity” characterized by high intensity but lower structural balance compared with international cultivars. PC2 (28.7%—Maturation vs. Fruitiness): The positive vertical axis was associated with maturation-related notes (Bit.E, Aft.E, Oak.E, and Dry.E), whereas the negative axis represented fresh varietal traits (Fru.E, Sw.E, and RtP.E). While CAB showed a balanced profile, SYR exhibited stronger oak and bitterness notes. BQ wines were consistently associated with deep purple hues (RtP.E) and high acidity. Following PCA and correlation analysis (|*r*| > 0.80), hierarchical feature selection was implemented to prevent overfitting in subsequent ANN modeling. The initial 23 attributes were streamlined into nine sensory traits (Table 2). These were categorized into the following:

- Primary variables (five attributes): RtP.E, Aro.E, So.E, Sw.E, and Tan.E, representing the “varietal identity core.”
- Supplementary variables (four attributes): Alc.E, Bit.E, Flaw.E, and Oak.E, representing vinification variance and quality risks.

To evaluate multicollinearity and establish a baseline for subsequent Artificial Neural Network (ANN) dependency analysis, the nine selected variables were initially subjected to a multiple linear regression model. Although the linear analysis identified critical drivers such as sourness (*p* < 0.001) and sensory flaw (*p* = 0.002) as statistically significant, the overall linear model fit was insufficient (*R*^2^ = 0.518), thereby justifying the transition to non-linear ANN modeling. Multicollinearity was strictly controlled throughout this feature selection stage, with all nine predictors exhibiting a variance inflation factor (VIF) of less than 2.0.

As quantitatively mapped in the PCA spatial distribution, the structural divergence between the international bridge samples and the Black Queen variants is mathematically explicit. The BQ samples consistently project onto the negative axis of PC1, defined by high varietal acidity and pigmentation, distinguishing them from the tannin-driven balance of the reference cultivars. This geometric separation confirms the necessity of establishing a cultivar-specific sensory baseline rather than relying solely on generalized international frameworks. Importantly, the inclusion of Cabernet Sauvignon and Syrah served as international sensory anchors rather than direct flavor comparators. As observed in Figure 2, these bridge samples effectively expand the principal component space, preventing the BQ coordinates from compressing. Furthermore, the close spatial clustering of the two independent Cabernet Sauvignon batches demonstrates the high consistency and low bias of the panel’s calibration.

### 3.3. Consumer Sensory Profiling via CATA and Penalty Analysis

To decode consumer perception, 52 initial CATA descriptors were evaluated by 74 consumers (*N* = 444 assessments). A rigorous filtration strategy was applied: descriptors were retained if their selection frequency exceeded 30% in at least one experimental block. Crucially, “risk descriptors” such as meaty and sweaty were manually retained regardless of frequency (<5%) based on the trained panel’s assessment of their high impact on wine quality. This process successfully streamlined the lexicon from 52 to 12 core CATA attributes. Penalty Analysis (mean drop plot) was employed to visualize the impact of these 12 attributes on the overall liking (OL) of Black Queen (BQ) wines (Table 2). The analysis identified six positive drivers and six negative factors:

- Positive Drivers (Mean Drop > 0): These attributes, including alcohol, violet, red berry, and blackberry, were associated with an increase in OL when perceived.
- Negative Factors (Mean Drop < 0): Attributes such as moldy, sulfur dioxide, tropical fruit and red plum significantly penalized the OL scores.

**Figure 3:**
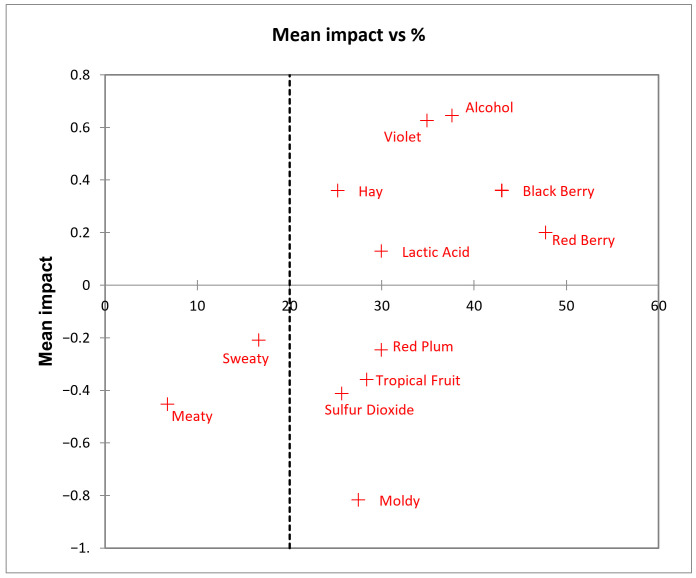
Penalty Analysis (mean drop) of CATA sensory attributes on consumer overall liking (OL). Note: Attributes in the upper-right quadrant represent positive drivers of liking, while those with significant negative mean drops are considered quality inhibitors (risks).

The resulting “mean drop vs. % consumer” plot (Figure 3) illustrates that while BQ-specific varietal markers (e.g., red plum and tropical fruit) have high frequency, their influence on quality judgment is mediated by the presence of process-related risks.

Table 2. The consolidated Lexicon v.0 functions as a highly filtered, low-collinearity input matrix for subsequent predictive modeling. By strategically classifying attributes into primary drivers, varietal markers, and high-impact risk factors, this architecture ensures that the neural network allocates weights to independent sensory dimensions, effectively mitigating the risk of tautological gradient inflation during training.

The mean drop topology visually quantifies the asymmetric impact of sensory traits on consumer overall liking. While baseline varietal markers exhibit high occurrence frequencies, their negative mean impacts underscore that consumer acceptance is disproportionately penalized by specific risk attributes, dictating a precision-driven approach to winemaking that prioritizes risk mitigation over mere aroma enhancement.

### 3.4. Finalization of Lexicon v.0 with Panel Decision

The integration of QDA and CATA results culminated in the establishment of Lexicon v.0, comprising 21 sensory traits (nine QDA and 12 CATA attributes). While six QDA attributes were retained based on robust statistical significance and high panel consensus, three additional attributes (Sw.E, Oak.E, and Bit.E) were included through panelist decision (PD) to ensure oenological completeness (Table 2 and Table S1). To optimize the predictive performance of the subsequent Artificial Neural Network (ANN), data pruning on Lexicon was implemented to eliminate tautology (redundancy). The input architecture was refined from a (9, 12) QDA and CATA configuration to a (7, 11) model. Specifically, two QDA variables (Alc.E and Flaw.E) and one CATA variable (sweaty/risk-related) were removed. This reduction was justified by the high semantic overlap: alcohol perception was better captured by the consumer CATA (Alc.C), while sensory flaws were more granularly represented by specific CATA risk factors (moldy, SO_2_, and meaty), thus preventing weight inflation within the neural network layers.

### 3.5. Predictive Modeling of Overall Liking via Artificial Neural Networks (ANNs)

The Artificial Neural Network (ANN) model utilized a (7, 11) configuration, integrating seven QDA descriptors and 11 CATA attributes as input variables (N = 18), with overall liking (OL) as the target output. To ensure the reliability of the 444 assessments, a five-fold K-fold validation method was employed with a fixed seed (888). The architecture featured two TanH and four Gaussian activation functions.

The model exhibited robust predictive performance (Table 3), with an *R*^2^ of 0.700 for the training set and 0.740 for the validation set. The root average squared error (RASE) remained stable at approximately 1.0, and the mean absolute error (MAE) was low (0.656 for validation), indicating that the model successfully avoided overfitting and possessed a high generalization capability [21,22].

Analysis of Variable Importance (Table 4) identified the primary drivers of consumer preference. Sourness (So.E) emerged as the most critical factor, with a total effect of 0.257, followed by sweetness (Sw.E) and blackberry (0.181). Notably, sourness (So.E) demonstrated an extraordinary interaction effect, where its total effect was approximately five times greater than its main effect, suggesting that acidity in Black Queen wines acts as a key driver that modulates the perception of all other flavor components.

The variable importance ranking derived from the ANN mathematically isolated sourness as the dominant regulatory node within the sensory network. The critical total-to-main effect ratio explicitly dictated that the influence of acidity operated predominantly through non-linear synergistic interactions with secondary traits, validating the deployment of advanced neural modeling over conventional linear regression for subtropical enology [10].

## 4. Discussion

The fundamental cornerstone of this study lies in the synchronization of two distinct data blocks using a Cabernet Sauvignon (CAB) bridge sample, which demonstrated excellent anchoring performance with no statistically significant differences (*p* > 0.05). To accommodate the unique phenotypic expressions of the rare Black Queen (BQ) hybrid, the CV threshold was set at < 30%, 10% wider than the normal width, considering BQ rarity and unfamiliarity [17,18], with Block 2 exhibiting consistently lower values than Block 1 due to the anchoring effect of two international benchmarks (CAB and SYR). Within Block 2, ten high-consensus attributes were directly integrated into the framework, seven were categorized as baseline noise, and six exhibited low consensus due to acid–sugar interactions or linguistic ambiguity. Through a deliberate panelist decision process, sweetness (Sw.E), oak (Oak.E), and bitterness (Bit.E) were expert-retained based on their diagnostic value and statistical support in the PCA. Although the resulting Lexicon v.0 initially comprised 21 sensory traits, three redundant variables overlapping between QDA and CATA were pruned to prevent neural network weight inflation. The optimized 18-input architecture was modeled using a single-layer, three-node Artificial Neural Network (ANN), achieving a robust coefficient of determination (*R*^2^ > 0.70) [21,22]. Global importance evaluation identified sourness (So.E), sweetness (Sw.E), blackberry, moldy, and aroma (Aro.E) as the primary drivers of consumer perception. Sourness emerged as the key driver of consumer acceptance, confirming that de-acidification is a mandatory baseline process requirement. Crucially, the total-to-main effect ratios for sourness, sweetness, and aroma reached 5.04, 3.61, and 3.04, respectively, demonstrating that non-linear, interactive effects far outweighed direct linear contributions. This extreme topographical complexity was vividly illustrated in the 3D response surfaces, where aroma exhibited a highly convoluted terrain, indicating an exceptionally narrow winemaking process window that demands precision oenological control.

### 4.1. Evaluation of Panel Consensus and Sensory “Bridge” Reliability

The primary objective of the “bridge” analysis was to validate the integration of independent data blocks into a unified database. The results confirm that the reference Cabernet Sauvignon sample successfully acted as a sensory anchor. Despite inherent individual variations among panelists, the statistical equivalence (*p* > 0.05) across all 23 descriptors confirms the compatibility of Block 1 and Block 2 (Table 1), providing a robust foundation for subsequent ANN (Artificial Neural Network) modeling. The analysis of low consensus and baseline noise categories provides critical insights into the limitations of human sensory perception and linguistic interpretation:

- The Low-Intensity Effect (Baseline Noise): High CV values (>80.0%) in attributes like mineral (Min.E) and bitterness (Bit.E) [23] are attributed to the low-intensity effect. In sensory science, small absolute differences at the lower end of the scale lead to large relative variations. These seven attributes (mean < 3.0) represent “baseline noise” and suggest that these specific notes were not prominent in the bridge sample.
- Cognitive and Linguistic Barriers (Low Consensus): The variance in attributes like vegetal (Veg.E) and floral (Flo.E) stems from the vast diversity of chemical compounds these terms encompass [24]. Without a single, universal reference standard, panelists may rely on different internal prototypes. Specifically, the herb (Her.E) attribute faced linguistic ambiguity in the Chinese context, where its semantic boundaries often overlap with vanilla, leading to divergence in scoring.
- Sweetness (Sw.E) and Aftertaste (Aft.E): The lack of consensus in sweetness was due to the interaction with high acidity in Black Queen wines, which complicates the perception of residual sugar [25,]. Aftertaste, being a composite and temporal attribute, lacks a physical standard and is subject to higher subjective interpretation.

Standard sensory evaluation protocols frequently employ a strict coefficient of variation (CV) ceiling of 20% to certify high panel consensus and data fidelity, a principle rigorously applied in both IOC-regulated olive oil tastings [17] and formal wine sensory quality audits [18]. While a CV exceeding 20% formally dictates sample re-evaluation due to panelist divergence [18], this study implemented an expanded threshold of 30% (Table 1). This threshold adjustment was specifically justified by the oenological novelty, sensory complexity, and cultural unfamiliarity surrounding the BQ cultivar. Relaxing the threshold slightly prevented the loss of emerging or latent sensory variables that are vital for capturing the unique varietal identity of BQ, while still filtering out baseline noise in accordance with robust sensometric practices.

We also observed that Block 2 exhibited generally lower CV values than Block 1. This could be attributed to the “anchoring effect” [11] provided by the inclusion of two distinct international benchmarks (Cabernet Sauvignon and Syrah) in Block 2, compared with the more repetitive BQ-focused design of Block 1. This suggests that a more diverse sample set during evaluation may improve panel calibration. In conclusion, the 10 high-consensus attributes identified are the most stable candidates for digital sensory transformation and future deep learning iterations (Table 1).

### 4.2. Dimensionality Reduction for Lexicon Refinement

In high-dimensional sensory data, multicollinearity (e.g., Sw.E/Fru.E; *r* = 0.93 in Table S2) often leads to “weight drowning” in machine learning, where redundant expert-defined intensities overwhelm the subtle variances in consumer liking.

- Elimination of Redundancy and Tautology: Attributes showing extreme collinearity (*r* > 0.90) and semantic overlap were pruned [26]. For instance, fruity (Fru.E) was replaced by sweetness (Sw.E, *r* = 0.93), and tannin coarseness (Cor.E) was merged into tannin (Tan.E, *r* = 0.94). Subjective composite terms like balance (Bal.E) with liking (*r* = 0.89) and Bal.E to Com.E (*r* = 0.92) were excluded; their inclusion would have introduced circular reasoning and data redundancy, as these terms are inherently evaluative rather than descriptive (Supplementary Table S2).
- The Tannin–Alcohol Paradox: A significant correlation was observed between tannin and alcohol (*r* = 0.87), suggesting that panelists may have conflated astringency with thermal sensation as a singular “body” dimension, leading to the confusion [27]. However, both were retained in the final nine-trait model. In oenological terms, alcohol (thermal) and tannins (phenolic) result from different viticultural and winemaking decisions; retaining both allows the ANN to differentiate between chemical structure and mouthfeel perception.
- Surrogates for Overall Liking: The strong positive correlation between aroma (Aro.E; *r* = 0.97) and overall liking (OL) suggests that BQ is essentially an “aromatic-driven” wine [28]. Conversely, the strong negative correlation with sourness (So.E; *r* = −0.91) confirms that the characteristic high acidity of the Black Queen cultivar remains a primary barrier to consumer acceptance [12].

By anchoring the PCA space (Figure 2) with international benchmarks (CAB and SYR), the unique “sensory fingerprint” of Black Queen wine—defined by high acid, deep pigment, and specific fermentation notes [12,13,14]—became statistically visible. This refined nine-attribute framework provides the necessary independence for the ANN classifier to accurately predict consumer responses without the interference of baseline noise or redundant semantic descriptors.

### 4.3. Decoding the Consumer Lexicon: Drivers, Varietal, Process, and Risks

The refinement of the CATA lexicon into 12 attributes allows for a multidimensional understanding of Black Queen wine quality. According to the mean drop (MD) values (Table 2) and oenological relevance, these attributes were categorized into four functional groups:

- Drivers of Liking (Must-Haves): Attributes like blackberry, red berry and floral exhibited positive MD values, indicating they are “must-have” traits (Figure 3) for consumer acceptance. For BQ wines, enhancing these fruit-driven factors is essential to counteract their inherent high acidity.
- Varietal Identifiers (BQ Markers): Red plum and tropical fruit notes emerged as high-frequency descriptors (>20%) uniquely associated with the BQ phenotype [12]. These markers define the “sensory typicity” of the cultivar but require careful balance to avoid being perceived as overly “wild” or “unrefined.”
- Process-Related Attributes: Traits like alcohol, hay, and lactic acid reflect the winemaking style. The positive MD associated with these notes suggests that consumers prefer BQ wines that have undergone controlled maturation, which may soften the aggressive sourness structure of the hybrid grape [12].
- Quality Risks (Deal-Breakers): Despite their low frequency, meaty, sweaty, and more common faults like moldy and sulfur dioxide showed severe negative MD values. This validates our “high-risk, low-frequency” retention strategy. In the context of Taiwan’s humid climate and small-scale vinification, these microbial and oxidative risks are critical “deal-breakers” that can negate the positive varietal attributes of Black Queen wine.

The interaction between these four categories highlights that the perceived quality of BQ wine is not just a sum of its parts, but a delicate avoidance of “risks” while maximizing its “varietal” and “driver” clusters. This 12-attribute CATA framework serves as a practical quality control tool for oenologists aiming to improve the marketability of Taiwanese hybrid wines.

### 4.4. Refinement of Lexicon v.0: Balancing Statistical Metrics with Oenological Reality

The construction of Lexicon v.0, comprising nine QDA attributes, highlights the necessity of balancing statistical rigor with expert oenological insight. While four attributes—aroma (Aro.E), sourness (So.E), tannin (Tan.E), and alcohol (Alc.E)—were directly integrated due to their robust statistical significance and semantic clarity, the remaining five (RtP.E, Sw.E, Oak.E, Bit.E, and Fla.E) exhibited more nuanced statistical characteristics [Tabel 2]. These attributes, whose relevance could not be definitively determined by automated inference alone, were retained through a deliberate panelist decision process. This expert intervention ensures the lexicon accounts for the psychophysical complexities of the Black Queen hybrid that purely objective filters might otherwise overlook.

- Sweetness (Sw.E) and the Acid–Sugar Masking Effect: Despite its low statistical consensus, sweetness was retained as a core driver [25,]. The observed variance is attributed to the acid–sugar masking effect, where the high titratable acidity of Black Queen grapes interferes with the sensory threshold for residual sugar. From a consumer perspective, sweetness follows an inverted-U curve (Optimal Arousal Theory) [29], acting as a critical hedonic driver. Overriding the statistical instability of Sw.E ensures the lexicon accounts for this non-linear relationship between acid–sugar balance and preference.
- Oak and Bitterness (Oak.E and Bit.E): In terms of industry relevance vs. risk monitoring, attributes like oak (Oak.E) and bitterness (Bit.E) were identified as baseline noise but were expert-retained for their diagnostic value [23]. Oak serves as an industry relevance marker, providing a benchmark for international quality standards. Conversely, bitterness was retained not as a driver of liking, but as a quality risk baseline. It monitors the physiological limit of phenolic extraction management; its inclusion allows Lexicon v.0 to diagnose over-extraction or fermentation imbalances that statistical averages might otherwise obscure.
- Typicity and Safety Baselines (RtP.E and Fla.E): The retention of red to purple (RtP.E) over-represents the varietal typicity of Black Queen [12,13,14]. While PCA (Figure 2) suggests it is an independent dimension, its low correlation with other flavor traits confirms it as a unique “visual fingerprint” of the cultivar. Similarly, off odor (Fla.E) was maintained as an aggregate safety baseline to monitor generic fermentation defects, providing a broader diagnostic layer than specific CATA terms.
- ANN Parsimony and Tautology Avoidance: The transition from 21 to 18 final ANN inputs (seven QDA and 11 CATA) addresses the challenge of multicollinearity-induced data redundancy. In neural network training, redundant variables—such as having both QDA and CATA versions of “alcohol”—can lead to overfitting and loss of feature importance, where the model over-emphasizes a single sensory dimension. By streamlining the inputs, we ensure the ANN maintains high sensitivity to diverse sensory signals while increasing the parsimony and generalizability of the quality prediction model.

### 4.5. Non-Linear Drivers of Consumer Preference and Model Robustness

The application of ANN in this study represents an advanced iteration of preference mapping. Unlike traditional linear regression [8,10], the ANN successfully captured the complex, non-linear interactions inherent in the sensory profile of hybrid wines like Black Queen [9].

- Overcoming Excessive Variables and Overfitting: A common challenge in sensory machine learning is the “curse of dimensionality,” where too many variables lead to sparse feature spaces. By implementing a four-stage mathematical refinement—reducing 23 initial QDA attributes to nine and finally to seven for ANN input—we maintained a high observation-to-variable ratio (~24.7:1). This far exceeds the heuristic “rule of thumb” (10–20:1), ensuring the model’s generalization ability and preventing weight explosion. The consistency between training and validation *R*^2^ (0.70 vs. 0.74) further confirms the model’s robustness [21,22].
- Tautology Management and Data Fusion: The strategic removal of redundant variables (e.g., deleting QDA Alc.E in favor of CATA alcohol) was essential to prevent data redundancy. This ensured that the neural network’s weights were assigned to unique sensory signals rather than being “drowned” by co-linear experts and consumer descriptors. This “clean” data fusion approach allowed the model to maintain a VIF of <3 across all inputs, significantly increasing the reliability of the importance rankings.
- Sourness as the Sensory Basis of Black Queen: The ANN importance results (Table 4) align perfectly with oenological reality. Sourness (So.E) was confirmed as the most determinant of overall liking. However, the discovery that its total effect (5.04) is five times its main effect provides a novel insight: acidity is not merely a taste attribute in BQ wine but a “modulator” that triggers non-linear interactions with sweetness [25,] and fruity notes (e.g., blackberry). This reflects the acid–sugar masking effect discussed earlier; acidity dictates the threshold at which other positive varietal markers can be perceived by consumers.
- Robustness and Varietal Identity: The consistent significance of the top five sensory attributes (So.E, Sw.E, Aro.E, blackberry and moldy) across various ANN configurations indicates that these traits may represent the core sensory markers defining the varietal identity of the Black Queen cultivar. From a viticultural and oenological perspective, these findings imply that acidity management extends beyond routine pH adjustments; rather, it plays a critical role in modulating the perception of aroma and sweetness, which are pivotal to consumer liking.

While non-linear interactions could theoretically be addressed via polynomial-PLS, expanding the feature space triggers a geometric explosion of terms (e.g., 10 variables generating 65 terms). This dimensional expansion renders iterative feature selection—relying on metrics such as AIC, VIP, and *Q^2^*—computationally impractical and prone to overfitting. As supported by Kirtil (2025) [10], the selected ANN architecture effectively bypasses this limitation, autonomously resolving complex non-linearities without explicit polynomial expansions.

### 4.6. ANN Visualization and Interaction Analysis for Precision Enology

**Figure 4:**
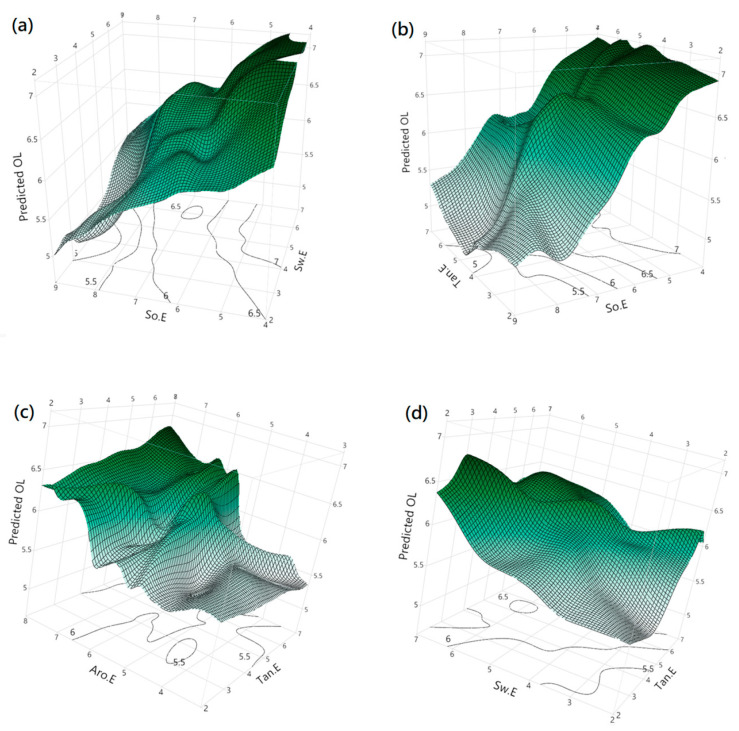
Three-dimensional response surface plots illustrating the non-linear interactions between key sensory attributes and consumer overall liking (OL): (**a**) interaction between sourness (So.E) and sweetness (Sw.E); (**b**) interaction between tannin (Tan.E) and sourness (So.E); (**c**) interaction between aroma (Aro.E) and tannin (Tan.E); and (**d**) interaction between sweetness (Sw.E) and tannin (Tan.E). Note: The 3D response surface plot represents a standardized global market projection. Artificial Neural Network (ANN) captures highly non-linear hyper-dimensional interactions to ensure reproducibility and eliminate bias; all non-axis sensory attributes were strictly anchored at their global sample means, representing the baseline marginal interactive effects. The green color gradient represents the magnitude of the Predicted OL, and the solid lines on the bottom plane are contour lines indicating constant Predicted OL values.

The visualization of the ANN model through interaction plots (Figure 4) provides additional interpretability for sensory data science by illustrating potential non-linear sensory landscapes in Black Queen (BQ) wines. ANN models are inherently data-driven and partially stochastic, resulting in some variation in response surface topology across repeated runs and parameter settings; several broader trends remained relatively consistent throughout the analyses. In most cases, sourness intensity (So.E) showed a generally negative association with overall liking (OL), suggesting that excessive sourness perception may reduce consumer acceptance [12]. In contrast, sweetness (Sw.E) tended to show a positive relationship with OL, although its influence was often less pronounced in regions dominated by strong So.E effects (Figure 4a) [30,31]. Compared with Figure 4c, aroma intensity (Aro.E) presented a more complex and irregular landscape, with stronger curvature and interaction behavior [24,32], implying that aroma-related optimization may involve highly non-linear sensory mechanisms that are difficult to regulate through simple process adjustments. In addition, several ANN surfaces suggested possible quadratic tendencies for tannin (Tan.E), with localized low-preference regions appearing at intermediate-intensity levels. Importantly, the interaction structures and local surface ruggedness appeared to be as meaningful as the overall positive or negative direction of individual variables. Relatively smooth regions may indicate broader and more manageable process windows, whereas highly curved or rugged regions suggest greater sensitivity to small sensory changes. Therefore, despite the inherent variability in ANN visualizations, the repeated emergence of similar directional tendencies supports the potential utility of ANN-based surface analysis as an exploratory tool for understanding sensory interactions and assisting precision-oriented enological optimization in BQ wines.

## 5. Conclusions

This research presents a structured sensory evaluation framework for Black Queen (BQ) wine through the development of Lexicon v.0 and a corresponding predictive ANN model. By employing a Cabernet Sauvignon “bridge” sample, this study demonstrated that two distributed data blocks could be effectively aligned, offering a viable approach for integrating sensory datasets into a larger machine learning big pool in oenology. Systematic dimensionality reduction using principal component analysis (PCA) and expert-led refinement streamlined 23 quantitative descriptive analysis (QDA) attributes into nine core traits. Concurrently, the inclusion of low-frequency yet critically high-risk defect descriptors—such as moldy, meaty, and sulfur dioxide—enhanced the lexicon’s utility for rigorous quality control tracking. The optimized ANN model, achieving an *R^2^* of 0.74 in the validation set, exhibited a higher explanatory power compared with traditional linear regression frameworks. Theoretically, this model identified five critical attributes, with sourness in Black Queen emerging as a key sensory determinant of overall consumer acceptance; specifically, the observed 5:1 total-to-main effect ratio underlines the complex, interactive role of acidity within the sensory matrix. Furthermore, the 3D interaction response surfaces suggested that BQ overall liking is governed by sourness, sweetness, and a complex, dynamic aroma. In practice, our findings establish the BQ Lexicon as a robust diagnostic and communication tool for winemakers. By identifying specific sensory determinants via the model (e.g., sourness), this framework allows winemakers to prioritize targeted quality management strategies. This transition from subjective evaluation to sensory-data-driven flavor engineering represents the primary practical contribution of this work. Regarding research limitations, we acknowledge that while the implemented ANN demonstrated predictive potential through strict dimensionality reduction and parsimonious architecture, applying machine learning to small-to-medium empirical sensory datasets (*N* = 444) remains inherently exploratory. Furthermore, the model’s reliance on a standardized bridge sample necessitates caution when generalizing results to broader, culturally diverse consumer populations. We explicitly stated that this framework is constrained by the regional nature of the Black Queen cultivar, specific vintages, and a shared cultural panel background. Future research should aim to expand the sample size through cross-institutional data fusion and integrate instrumental analysis to correlate chemical markers with the current sensory lexicon, thereby evolving this framework into a comprehensive “Lexicon v.1” for precision enology. Ultimately, the integration of trained panel QDA, consumer check-all-that-apply (CATA) analysis, and ANN modeling provides a potential methodological framework for sensory evaluation and flavor engineering in winemaking.

## Figures and Tables

**Table 1:** Inter-Site Consistency Analysis (complete 23 QDA attributes).

Category	Attribute	Code	Block 1 (*n* = 11)	B1 CV	Block 2 (*n* = 11)	B2 CV	*p*-Value	Consensus Status
Appearance	Color depth	Dep.E	7.02 ± 1.10	15.70%	7.04 ± 0.76	10.80%	0.960	Consensus
	Red to Purple	RtP.E	6.99 ± 0.74	10.60%	7.21 ± 0.33	4.60%	0.382	Consensus
Aroma	Off odor	Fla.E	1.32 ± 2.00	151.60%	1.19 ± 1.01	85.20%	0.853	Baseline noise
	Aroma	Aro.E	5.79 ± 1.91	32.90%	6.00 ± 1.20	20.00%	0.772	Consensus
	Sweetness	Sw.E	5.38 ± 2.44	45.50%	5.46 ± 1.89	34.70%	0.935	Low consensus
	sourness	So.E	6.50 ± 1.68	25.80%	6.72 ± 0.56	8.40%	0.677	Consensus
	Fruity	Fru.E	6.78 ± 1.61	23.70%	7.25 ± 0.86	11.80%	0.414	Consensus
	Floral	Flo.E	4.19 ± 2.73	65.10%	3.35 ± 2.25	67.20%	0.461	Low consensus
	Spicy	Spi.E	6.00 ± 2.43	40.50%	4.90 ± 1.69	34.40%	0.249	Low consensus
	Herb	Her.E	3.56 ± 2.46	69.10%	3.03 ± 1.42	46.80%	0.551	Low consensus
	Vegetal	Veg.E	5.98 ± 2.25	37.70%	4.52 ± 2.51	55.40%	0.191	Low consensus
	Dried plant	Dry.E	2.09 ± 1.99	95.50%	2.56 ± 2.16	84.10%	0.618	Baseline noise
	Caramel	Car.E	1.91 ± 2.30	120.60%	2.32 ± 2.01	86.70%	0.671	Baseline noise
	Animal	Ani.E	1.77 ± 2.34	131.90%	2.17 ± 2.12	97.40%	0.693	Baseline noise
Taste and MF	Mineral	Min.E	2.95 ± 3.09	104.50%	2.30 ± 2.36	102.90%	0.595	Baseline noise
	Oak	Oak.E	1.90 ± 2.26	118.70%	2.60 ± 1.94	74.80%	0.468	Baseline noise
	Tannin	Tan.E	6.83 ± 1.30	19.00%	5.99 ± 1.67	28.00%	0.235	Consensus
	Coarseness	Cor.E	6.58 ± 1.74	26.50%	6.59 ± 0.62	9.30%	0.980	Consensus
	Alcohol	Alc.E	7.06 ± 1.07	15.20%	6.58 ± 0.83	12.60%	0.276	Consensus
Overall	Balance	Bal.E	5.23 ± 2.42	46.20%	5.85 ± 0.72	12.30%	0.421	Consensus
	Complexity	Com.E	3.65 ± 2.15	59.10%	4.65 ± 1.05	22.60%	0.191	Consensus
	Finish	Aft.E	4.71 ± 2.67	56.80%	4.99 ± 1.39	27.80%	0.768	Low consensus
	Bitterness	Bit.E	2.31 ± 3.07	132.60%	2.91 ± 2.48	85.30%	0.637	Baseline noise

Note: Mean ± SD (*n* = 11); *p* > 0.05 (independent *t*-test) denotes no significant difference between blocks. CV% = (SD/Mean) × 100. Status: Consensus on B2 (CV < 30%), low (30–70%), and baseline noise (mean < 3.0 and CV > 70%).

**Table 2:** Sensory Lexicon (v.0) for Black Queen wine: 21 defined descriptors.

	Category	Attribute	Code	Status	VIF	Sensory Reference Standards	Remark
**QDA**	Appearance	Red to purple	RtP.E	Primary	1.380	Pantone: 19-1629 TCX (Ruby Wine), 19-1522 TCX (Mahogany)	Defines the visual baseline for BQ varietal identity
	Aroma	Overall aroma	Aro.E	Primary	1.540	Diverse profile; no standards yet	Foundational intensity metric for general quality
	Taste	Sweetness	Sw.E	Primary	1.380	Sucrose solution, 24.0 g/L (ISO 3972 [19])	Vital modulator for balancing BQ’s naturally high acidity
		Sourness	So.E	Primary	1.150	Citric acid solution, 1.2 g/L (ISO 3972)	Hallmark BQ varietal trait; high statistical independence
	Mouthfeel	Tannin	Tan.E	Primary	1.260	Tannic acid solution, 0.5 g/L (ISO 8586 [20])	Core structural component representing oral friction
	Processing	Alcohol	Alc.E	Suppl.	1.390	Standardized 12% *v*/*v* ethanol/water matrix	Reflects perceived body, warmth, and fermentation success
		Bitterness	Bit.E	Suppl.	1.700	Diverse profile; no standards yet	Secondary structural trait; indicator of seed/stem extraction
		Oak	Oak.E	Suppl.	1.870	Diverse profile; no standards yet.	Key indicator for maturation and processing techniques
		Off odor (fault)	Flaw.E	Suppl.	1.500	Diverse profile; no standards yet	Critical marker for process quality and risk assessment
	**Category**	**Sensory Trait**	**Code**	**Mean** **Impact**	*p* **-Value**	**Sensory Reference Standards**	**Remark**
**Hedonic (CATA)**	Drivers	Alcohol	Alc.C	0.645	<0.001 *	10 mL of Binggrae Banana Milk (commercial product)	Primary structural driver of liking
		Violet	Vio.C	0.626	<0.001 *	Diverse profile; no standards yet	Key floral positive driver
		Blackberry	BBe.C	0.360	0.022 *	5 mL of Crème de Cassis de Dijon (20% ABV, Reflets de France)	Baseline fruitiness for red wine
	Varietal	Tropical fruit	Tro.C	−0.360	0.038 *	10 g of Crushed Taiwanese Red Plum (local fresh fruit)	Signature BQ trait; requires non-linear balancing
		Red plum	Plu.C	−0.247	0.149	10 mL of Ocean Spray 100% cranberry juice	Varietal marker; latent variable for synergy
		Red berry	RBe.C	0.200	0.202	Standardized 12% *v*/*v* ethanol/water matrix	Background fruitiness profile
	Process	Lactic acid	Lac.C	0.129	0.451	10 mL of plain yogurt filtrate (aqueous phase)	Critical for acid balance; MLF indicator
		Hay	Hay.C	0.359	0.046 *	1 g of dried rice straw powder (sun-dried and pulverized)	Secondary aromatic complexity
	Risks	Moldy	Mol.C	−0.817	<0.001 *	Not utilized for safety	Process killer; severe negative weight
		Sulfur dioxide	SO2.C	−0.413	0.021 *	Not utilized for safety	Management indicator; significant penalty
		Sweaty	Swe.C	−0.209	n.s.	5% (*w*/*v*) MRS broth solution	Brettanomyces risk; low frequency but high impact
		Meaty	Mea.C	−0.453	n.s.	Aromathèque Reference #No. 101 (horse sweat) + No. 92 (yogurt)	High-risk killer; non-linear penalty marker

Note: QDA, Quantitative Descriptive Analysis; CATA, check-all-that-apply; VIF, variance inflation factor. Status indicates the variable selection tier (primary: core model predictors; suppl.: supplementary descriptors). Mean impact represents the change in consumer overall liking (OL) score when the attribute is selected. * indicates statistically significant impact on liking (*p* < 0.05); n.s., not significant (*p* ≥ 0.05). “Not utilized for safety” indicates that physical reference standards were omitted during panel training due to toxicity, inhalation risks, or chemical safety protocols.

**Table 3:** ANN model performance (top 10).

Dataset	*n*	*R* ^2^	RASE	Mean Abs. Dev. (MAE)
Training set	355	0.700	1.069	0.689
Validation set	89	0.740	1.011	0.656

**Table 4:** Variable importance of sensory attributes (dependent resample inputs).

Rank	Sensory Attribute	Main Effect	Total Effect	Total/Main Ratio	Remark
1	Sourness (So.E)	0.051	0.257	5.04	Key structural driver
2	Sweetness (Sw.E)	0.051	0.184	3.61	Perceptual gatekeeper
3	Black berry	0.052	0.181	3.48	Framework modifier
4	Moldy	0.053	0.157	2.96	Positive aromatic driver
5	Aroma (Aro.E)	0.051	0.155	3.04	Negative quality marker
6	Red berry	0.067	0.143	2.13	Fruit complement
7	Alcohol	0.067	0.13	1.94	Balance contributor
8	Violet	0.069	0.129	1.87	Varietal character
9	Tropical fruit	0.057	0.124	2.18	Varietal character
10	Red plum	0.046	0.124	2.70	Complexity factor

## Data Availability

The data presented in this study are available upon request from the corresponding author. The data are not publicly available at this time due to the ongoing finalization of the version sensory lexicon (v1.0) for Black Queen.

## Supplementary Materials

The following supporting information can be downloaded at https://www.mdpi.com/article/10.3390/foods15122158/s1: Table S1. Pruning rationale for 23 QDA attributes; Table S2. Correlation matrix between 23 QDA attributes and overall liking (OL).
