# Supplementary material for: Development of a Sensory Lexicon and Predictive ANN Modeling for Black Queen Wine: A Novel Workflow Incorporating Bridge-Linked QDA and Consumer Hedonic Analysis
Source: Foods. 2026 Jun 15;15(12):2158. doi: 10.3390/foods15122158 (PMC13298715; doi:10.3390/foods15122158)
Supplement: Supplementary file 1 [file foods-15-02158-s001.zip › Table S1. Pruning Rationale for 23 QDA Attributes.pdf]

Supplementary Table S1. Pruning Rationale for 23 QDA Attributes

Attribute	Code	Category	Lexicon	Consensus Status	Pruning Rationale & Statistical Basis	Panel Conclusion
Color Depth	Dep.E	Appearance	Delete	Consensus	Replaced by RtP.E (Red to Purple); BQ characteristic.	Tautological Convergence: Replaced by RtP.E for better typicity representation.
Red to Purple	RtP.E	Appearance	Keep	Consensus	Key discriminator for Black Queen; retained for specificity.	Panelist Decision: Superior discriminator for Black Queen; consensus on visual specificity.
Off-odor	Fla.E	Aroma	Keep	Baseline Noise	Retained to monitor low-frequency high-risk flaws.	No Consensus, But it's vital safety baseline; monitors fermentation-derived flaws.
Total Aroma	Aro.E	Aroma	Keep	Consensus	Essential indicator of sensory intensity.	Consensus: Essential indicator of overall sensory intensity and impact.
Sweetness	Sw.E	Taste	Keep	Low consensus	Core taste driver.	Panelist Decision: Critical driver in Wundt Curve; masking by high acidity reduced consensus.
Sourness	So.E	Taste	Keep	Consensus	Core taste driver; BQ hallmark.	Consensus: Fundamental hallmark of Black Queen varietal character.
Fruity	Fru.E	Aroma	Delete	Consensus	Redundant; strong correlation with Sw.E r=0.92.	Tautological Convergence: Highly redundant with Sw. E r=0.92; aroma-induced sweetness.
Floral	Flo.E	Aroma	Delete	Low Consensus	Low explained variance in PCA; poor panel consistency.	Low Consensus: Low explained variance in PCA; inconsistent panel perception.
Spicy	Spi.E	Aroma	Delete	Low Consensus	Redundant with Tan.E & Cor.E in PCA clusters.	Low Consensus: PCA showed clustering with Tan.E and Cor.E.
Herbaceous	Her.E	Aroma	Delete	Low Consensus	Semantic divergence in Chinese; caused panel confusion.	Low Consensus: Semantic divergence in Chinese; caused panelist divergence.
Vegetal	Veg.E	Aroma	Delete	Low Consensus	Excessive noise; weak correlation with core drivers.	Low Consensus: Weak correlation with core drivers; high background noise.
Dried Plant	Dry.E	Aroma	Delete	Baseline Noise	Redundant with Oak.E r=0.92; semantic overlap.	Highly Tautological: Conceptual overlap and high correlation with Oak.E r=0.92.
Caramel	Car.E	Aroma	Delete	Baseline Noise	Redundant with Oak.E r=0.81.	Highly Tautological: Redundant with Oak.E r=0.81; semantic similarity.
Animal	Ani.E	Aroma	Delete	Baseline Noise	Highly redundant with Fla.E r=0.97.	Highly Tautological: Functionally identical to Fla.E r=0.97 in PCA space.
Mineral	Min.E	Taste	Delete	Baseline Noise	Replaced by Fla.E/Ani.E cluster r=0.97.	Baseline Noise: Redundant with Fla.E/Ani.E r=0.97; lacks independent impact.
Oak	Oak.E	Aroma	Keep	Baseline Noise	International standard; retained for industry relevance.	Panelist Decision: Critical international benchmark; retained despite high variance.
Tannin	Tan.E	Mouthfeel	Keep	Consensus	Core structural driver.	Consensus: Primary structural driver for red wine mouthfeel profiling.
Coarseness	Cor.E	Mouthfeel	Delete	Consensus	Redundant with Tan.E r=0.94; semantic overlap.	Consensus: Independent structural factor; distinct from tannin intensity r=0.85.
Alcohol	Alc.E	Mouthfeel	Keep	Consensus	Independent structural factor r=0.85 vs Tan.E).	Consensus: Retained as an independent structural factor r=0.85 vs Tan.E).
Balance	Bal.E	Overall	Delete	Consensus	Composite factor; high redundancy with Aro.E r=0.92.	Highly Tautological: Composite factor; high redundancy with Aro.E r=0.92.
Complexity	Com.E	Overall	Delete	Consensus	Subjective & cross-correlated with multiple attributes.	Highly Tautological: Subjective summation of primary drivers; high interaction effects.
Finish	Aft.E	Overall	Delete	Low Consensus	Subjective; redundant with Bit.E r=0.90.	Highly Tautological: Overlap with Bit.E r=0.90; subjective persistence issues.
Bitterness	Bit.E	Taste	Keep	Baseline Noise	Retained for quality risk monitoring.	Panelist Decision: Essential quality-risk baseline; monitors extraction management.
