# Supplementary material for: Development of a Sensory Lexicon and Predictive ANN Modeling for Black Queen Wine: A Novel Workflow Incorporating Bridge-Linked QDA and Consumer Hedonic Analysis
Source: Foods. 2026 Jun 15;15(12):2158. doi: 10.3390/foods15122158 (PMC13298715; doi:10.3390/foods15122158)
Supplement: Supplementary file 1 [file foods-15-02158-s001.zip › Table S2. Correlation Matrix between 23 QDA Attributes and Overall Liking (OL).pdf]

Supplementary Table S2. Correlation Matrix between 23 QDA Attributes and Overall Liking (OL)

	**OL**	Dep.E	**RtP.E**	Flaw.E	Aro.E	Sw.E	So.E	**Fru.E**	Flo.E	Spi.E	**Her.E**	Veg.E	Dry.E	Car.E	**Anim.E**	Min.E	Oak.E	Tan.E	Cor.E	Alc.E	**Bal.E**	Com.E	Aft.E	Bit.E
**OL**	1.00	0.00	-0.46	-0.79	0.97	0.48	-0.91	0.50	0.64	0.57	-0.21	0.80	0.23	0.81	-0.87	-0.39	0.45	0.27	0.07	0.34	0.89	0.69	-0.14	0.06
Dep.E	0.00	1.00	-0.38	-0.50	0.06	-0.29	0.32	0.01	-0.64	0.71	-0.45	0.08	0.55	0.17	-0.46	-0.64	0.47	0.83	0.75	0.74	0.33	0.37	0.20	0.09
RtP.E	-0.46	-0.38	1.00	0.62	-0.57	0.54	0.31	0.46	-0.13	-0.73	-0.18	-0.18	-0.91	-0.84	0.65	0.13	-0.97	-0.70	-0.53	-0.33	-0.79	-0.88	-0.61	-0.84
Flaw.E	-0.79	-0.50	0.62	1.00	-0.77	-0.13	0.48	-0.34	-0.04	-0.92	0.52	-0.79	-0.43	-0.76	0.97	0.70	-0.59	-0.75	-0.59	-0.76	-0.87	-0.67	0.16	-0.09
Aro.E	0.97	0.06	-0.57	-0.77	1.00	0.33	-0.91	0.38	0.62	0.57	-0.18	0.65	0.33	0.91	-0.88	-0.42	0.57	0.29	0.03	0.25	0.92	0.77	0.05	0.21
Sw.E	0.48	-0.29	0.54	-0.13	0.33	1.00	-0.52	0.93	0.46	-0.13	-0.31	0.59	-0.59	-0.10	-0.19	-0.19	-0.48	-0.36	-0.36	0.09	0.09	-0.15	-0.68	-0.75
So.E	-0.91	0.32	0.31	0.48	-0.91	-0.52	1.00	-0.44	-0.87	-0.21	-0.03	-0.57	-0.07	-0.73	0.62	0.13	-0.31	0.10	0.31	0.06	-0.71	-0.57	0.03	-0.10
Fru.E	0.50	0.01	0.46	-0.34	0.38	0.93	-0.44	1.00	0.21	0.09	-0.61	0.65	-0.53	-0.02	-0.37	-0.53	-0.40	-0.13	-0.18	0.30	0.18	-0.12	-0.73	-0.81
Flo.E	0.64	-0.64	-0.13	-0.04	0.62	0.46	-0.87	0.21	1.00	-0.17	0.46	0.29	-0.01	0.45	-0.21	0.36	0.15	-0.41	-0.52	-0.38	0.41	0.39	0.10	0.18
Spi.E	0.57	0.71	-0.73	-0.92	0.57	-0.13	-0.21	0.09	-0.17	1.00	-0.37	0.62	0.67	0.65	-0.88	-0.59	0.73	0.94	0.83	0.87	0.81	0.73	0.05	0.27
Her.E	-0.21	-0.45	-0.18	0.52	-0.18	-0.31	-0.03	-0.61	0.46	-0.37	1.00	-0.43	0.33	-0.06	0.43	0.93	0.23	-0.29	-0.20	-0.48	-0.12	0.19	0.62	0.61
Veg.E	0.80	0.08	-0.18	-0.79	0.65	0.59	-0.57	0.65	0.29	0.62	-0.43	1.00	-0.01	0.44	-0.75	-0.44	0.15	0.38	0.35	0.66	0.64	0.36	-0.59	-0.31
Dry.E	0.23	0.55	-0.91	-0.43	0.33	-0.59	-0.07	-0.53	-0.01	0.67	0.33	-0.01	1.00	0.60	-0.47	0.00	0.96	0.74	0.63	0.39	0.64	0.85	0.75	0.84
Car.E	0.81	0.17	-0.84	-0.76	0.91	-0.10	-0.73	-0.02	0.45	0.65	-0.06	0.44	0.60	1.00	-0.84	-0.35	0.80	0.46	0.19	0.22	0.93	0.86	0.34	0.55
Anim.E	-0.87	-0.46	0.65	0.97	-0.88	-0.19	0.62	-0.37	-0.21	-0.88	0.43	-0.75	-0.47	-0.84	1.00	0.67	-0.65	-0.68	-0.46	-0.65	-0.95	-0.77	0.04	-0.15
Min.E	-0.39	-0.64	0.13	0.70	-0.42	-0.19	0.13	-0.53	0.36	-0.59	0.93	-0.44	0.00	-0.35	0.67	1.00	-0.12	-0.49	-0.31	-0.56	-0.41	-0.16	0.33	0.34
Oak.E	0.45	0.47	-0.97	-0.59	0.57	-0.48	-0.31	-0.40	0.15	0.73	0.23	0.15	0.96	0.80	-0.65	-0.12	1.00	0.72	0.55	0.37	0.80	0.94	0.68	0.82
Tan.E	0.27	0.83	-0.70	-0.75	0.29	-0.36	0.10	-0.13	-0.41	0.94	-0.29	0.38	0.74	0.46	-0.68	-0.49	0.72	1.00	0.94	0.87	0.62	0.63	0.18	0.34
Cor.E	0.07	0.75	-0.53	-0.59	0.03	-0.36	0.31	-0.18	-0.52	0.83	-0.20	0.35	0.63	0.19	-0.46	-0.31	0.55	0.94	1.00	0.88	0.40	0.43	0.06	0.24
Alc.E	0.34	0.74	-0.33	-0.76	0.25	0.09	0.06	0.30	-0.38	0.87	-0.48	0.66	0.39	0.22	-0.65	-0.56	0.37	0.87	0.88	1.00	0.51	0.39	-0.27	-0.13
Bal.E	0.89	0.33	-0.79	-0.87	0.92	0.09	-0.71	0.18	0.41	0.81	-0.12	0.64	0.64	0.93	-0.95	-0.41	0.80	0.62	0.40	0.51	1.00	0.92	0.20	0.40
Com.E	0.69	0.37	-0.88	-0.67	0.77	-0.15	-0.57	-0.12	0.39	0.73	0.19	0.36	0.85	0.86	-0.77	-0.16	0.94	0.63	0.43	0.39	0.92	1.00	0.53	0.66
Aft.E	-0.14	0.20	-0.61	0.16	0.05	-0.68	0.03	-0.73	0.10	0.05	0.62	-0.59	0.75	0.34	0.04	0.33	0.68	0.18	0.06	-0.27	0.20	0.53	1.00	0.88
Bit.E	0.06	0.09	-0.84	-0.09	0.21	-0.75	-0.10	-0.81	0.18	0.27	0.61	-0.31	0.84	0.55	-0.15	0.34	0.82	0.34	0.24	-0.13	0.40	0.66	0.88	1.00

2026/5/22
